# Effects of Infection Control Education for Nursing Students Using Standardized Patients vs. Peer Role-Play

**DOI:** 10.3390/ijerph18010107

**Published:** 2020-12-26

**Authors:** Eunyoung Kim, Sang Suk Kim, Sunghee Kim

**Affiliations:** 1 College of Nursing, Yonsei University, Seoul 03722, Korea; key7481@daum.net; 2 Red Cross College of Nursing, Chung-Ang University, Seoul 06974, Korea; sung1024@cau.ac.kr

**Keywords:** infection control, nursing student, knowledge, awareness, education

## Abstract

This study was conducted to identify and compare the effects of two education programs for infection control―a simulation using standardized patients and a peer role-play―on standard precaution knowledge, standard precaution awareness, infection-related anxiety, and infection control performance. This study used a nonequivalent control group pretest-posttest design. A total of 62 undergraduate nursing students in their 3rd year participated in the study, and were assigned to the experimental and control groups, accordingly. The infection control education program was developed based on the analysis, design, development, implementation, and evaluation model. The program for the experimental group included lectures, skills training, simulation using standardized patients, and debriefing, while the control group participated in the usual infection control education, consisting of lectures, skills training, and peer tutoring practices. Both groups exhibited statistically significant increases in knowledge, awareness of standard precaution, and infection control performance after the intervention. Infection-related anxiety and infection control performance were significantly higher in the simulation using a standardized patient group. Both education programs influenced compliance with the standard precaution for infection control. The results of this study contribute to the evidence regarding effective educational methods to improve infection control.

## 1. Introduction

Healthcare-related infections impair an individual’s quality of life due to increased pain and mortality, deteriorated health, dysfunction, cognitive impairment, and financial loss of the health management system because of delayed recovery and extended hospital stay [1,2]. Moreover, the World Health Organization (WHO) announced the existence of the coronavirus disease 2019 (COVID-19), which is rapidly spread and threatens hospitals worldwide and public health as a whole [3]. The WHO’s Practical guidelines for infection control in health care facilities [4] require the isolation of individuals with severe acute respiratory syndrome, new influenza infecting humans, and new respiratory diseases that can potentially have a significant public health impact. Awareness, knowledge, and the attitudes in health care professionals’ practice are critical in managing viral diseases [5].

Standard precaution, the most basic management activity to prevent infection, was first announced in 1996 by the Centers for Disease Control and Prevention (CDC). A revised version followed in 2007, due to the emergence of new pathogens, such as Severe Acute Respiratory Syndrome, and multiple outbreaks involving blood-borne viruses (BBVs) resulting from injection practices [6]. More recently, the CDC recommended changes to the guidelines in view of the 2019 coronavirus pandemic [7]. The standard precaution guidelines are based on existing scientific data and evidence gathered through a comprehensive literature review [8,9,10,11], and the recommendations are classified into five stages [6]. Standard precaution is considered to be the most effective way to prevent healthcare-related infections among both patients and healthcare workers [12], and healthcare professionals are strongly advised to adhere to these practices. A systematic review of the literature reported that compliance with standard precaution improves patient safety, prevents healthcare-related infections, and contributes to healthcare workers’ safety [13]. 

Standard precaution requires healthcare professionals to consider all patient secretions to prevent the spread of pathogens, including blood and body fluids, as potential infection sources, and avoid being exposed to them; it is recommended that people should comply with this regarding any infection before the disease is diagnosed [6]. In addition, the WHO recommends applying the practice of standard precaution among all patients, including practicing hand and respiratory hygiene, using appropriate personal protective equipment (PPE) according to risk assessment as well as proper linens, promoting injection safety practices and safe waste management, environmental cleaning, and sterilizing patient-care equipment [14]. Moreover, the CDC recommends that hospitals and health institutions provide infection control education to patients and healthcare providers and visitors to and from the hospitals and trainees of medical and nursing schools, using the standard guidelines for medical-related infection [15]. 

Education on infection control improves health professionals’ adherence to standard precautions [16]. For instance, teaching infection control in nursing education is valuable for preventing nosocomial infection and reducing the infection rate. As healthcare professionals, nurses worldwide are on the front lines in caring for infected patients [17]. Furthermore, as future healthcare professionals, nursing students make up the most considerable portion of every country’s health workforce. Based on the nursing curriculum, nursing students must perform clinical practice, wherein a significant portion of their time is spent in contact with patients. During this period, nursing students are exposed to a higher risk of exposure to infectious agents compared to nurses [18], due to their lack of experience in clinical techniques and preparing for safety accidents [19]. Thus, infection control education is critical in laying the foundation for nursing college students to improve their infection control practical skills, which is required in the clinical field [20,21]. Al-Hussami and Darawad [22] suggested that the best way to improve nurses’ infection control activities is to educate nursing college students throughout the undergraduate program in performing and practicing infection control techniques from the beginning of their clinical practice. Ultimately, well-educated nursing students may affect the quality of care provision.

Previous studies with nursing students examining the level of awareness and performance of standard precaution argue that continuous and repetitive practical training of standard precaution needs to be supplemented for more efficient education [18,23]. In clinical settings, nursing students do not have sufficient clinical experience due to patients’ demands for high-quality nursing and medical services from health professionals [24,25]. Thus, it is necessary to develop and apply appropriate educational methods that can be used in clinical practice, and not just lecture-style education that focuses on simple knowledge transfer [26,27].

Simulation-based education is a field-reproducible method of teaching that uses an environment similar to the actual clinical situation. It allows for safe and effective repeated learning, as desired [28,29]. Besides, conducting a simulation using standardized patients (SP) who are well-trained in expressing their disease and emotional state, similar to the real world, would allow students to realize the patient’s thoughts and feelings by interacting with them and have more realistic and concrete experiences [30]. Another practical education method is peer role-play (PRP), which is a low-cost tool that can be quickly incorporated into practice [31]. It allows for the switching of roles to experience both health provider and patient perspectives. The use of SP and PRP constitute the main components of medical and nursing education [31,32]. However, there is a lack of empirical studies comparing simulation-based and PRP methods of educating nursing students on standard precautions for infection control. Moreover, the Korea Centers for Disease Control and Prevention and the Korean Society for Health Care-associated Infection Control and Prevention developed a guideline for standard precautions for healthcare-related infection prevention in 2017 [15]. There are even fewer studies that have examined the effectiveness of standard precaution infection control education among Korean nursing students.” Therefore, the current study may add empirical evidence for standard precaution to the existing literature. It will also provide useful educational methods for standard precautions for nursing practical education, reducing the risk of transmission of viruses and other pathogens in hospital settings.

This study aimed to grasp the effects of two education methods: (1) simulation-based education using SP, and (2) usual education using PRP, and their application in standard precaution infection control education for nursing college students.

## 2. Materials and Methods

### 2.1. Study Design

A nonequivalent control group pretest-posttest design was used to compare the effects of simulation using SP and PRP education for nursing college students.

### 2.2. Participants

Among the 288 nursing students in their third year at C University, we included nursing students who (1) were interested in infection control education, (2) understood the purpose of the study and expressed their intention to participate voluntarily, and (3) were conveniently extracted on a first-come, first-served basis. Previous research has suggested that it is necessary to develop a module for intermediate-level students to practice infection control skills within clinical scenarios before proceeding with complex scenarios for senior-year students or graduate nurses [33]. Accordingly, the participants were students who were in the third year, which marks the beginning of their clinical practice.

The number of samples for the study was calculated using the G*Power 3.1.9.4 program [34], with a significance level (α) of 0.05, effect size (*d*) of 0.8, and power (1-β) of 0.80. The effect size was in line with effect sizes used in previous studies’ effect sizes [35,36]. According to the results, a total of 52 patients were required, with 26 patients each in the intervention and control groups. In this study, 3 out of 65 participants dropped out (drop rate 4%); hence, there were 29 and 33 participants in the intervention and control groups, respectively.

### 2.3. Program Development

The development of the infection control education program using SP in this study is based on the analysis, design, development, implementation, and evaluation (ADDIE) model, which is designed for the education system. For the analysis stage, the educational needs, targeted learner (optimal target grade for the program before starting clinic practice), and the education environment were analyzed using a questionnaire regarding the need for infection control education on standard precautions for graduate nurses. Clinical scenarios suitable for 3rd year level nursing students were selected, and an educational environment that could reproduce clinical trials was analyzed.

In the design stage, to formulate educational goals, contents, and strategies, we set evaluation strategies to achieve the educational goals on knowledge, skills, and attitudes with regard to the infection control program. In addition, simulation education was selected as the educational medium. Standard precautions were defined and composed according to their educational content for each element and propagation path, as well as the simulation education and evaluation applying standard precautions. The development stage was conducted to develop the educational content by modifying and supplementing the program based on the pilot study. In this study, content validity was verified by correcting the scenarios to which the standard precautions were applied. Thereafter, SP training and simulation trial operation was conducted.

In the implementation stage, we applied the developed program accordingly. In this study, 29 nursing students participated in simulation education using SP. In addition, they participated in debriefing after the simulation to share their experiences. Meanwhile, 33 nursing students participated in usual practical training using PRP. The evaluation stage was conducted to determine the appropriateness of a systematic and cyclical education program. In this study, the knowledge and awareness of standard precautions as well as the ability to perform infection control was measured to evaluate the effectiveness of the standard precautions infection program.

### 2.4. Procedure of the Program

To evaluate the effectiveness of the infection control education program, participants were classified in either of the two groups: (1) those who will undergo simulation using SP, or (2) those who will undergo PRP practice after receiving a lecture about infection and nursing skills related to infection performance (Table 1).

The lecture was conducted using a PowerPoint presentation provided by the researcher, which included the definition and components of standard precautions as well as the attention of each propagation path. Skill training included performing subcutaneous injection, promoting hand hygiene, using PPE (including N95 masks and gloves), and finger-stick glucose monitoring related to nursing intervention; these were then applied to the simulation scenario.

Simulation using SP was conducted in the order of simulation orientation, simulation operation, and debriefing. During the orientation, we explained to the participants the study’s learning objectives, scenario situation, and simulation environment. In addition, we provided them with sufficient time to confirm the location of the items to be prepared and to familiarize themselves with the simulation environment. The simulation practice was conducted as follows: (1) participants wore their PPE before entering the simulated isolation room outside the patient’s room; (2) they assessed the patient’s condition; (3) they performed proper nursing practices, such as monitoring vital signs, checking blood sugar, and performing subcutaneous injection; (4) they exited the hospital room and took off their PPE. A total of seven teams participated, with each team consisting of four students that were equally assigned to two groups (two students per group)—one group participated in the simulation while the other observed the simulation, and vice versa. The running time of the complete simulation was 180 minutes.

Debriefing was conducted in the Description-Analysis-Application stage [37]. During this period, the students looked back on the simulation in the description stage and determined their overall feeling of the process as a whole. In the analysis stage, each individual’s experience while practicing as well as their strengths and weaknesses in the performance of nursing-related tasks were analyzed. In the application stage, the future clinical application methods and lessons learned through simulation were shared.

The two SPs who participated in the simulation belong to the simulation center of C University and have participated in many simulation training. The patients in the scenario are in their 60s to 70s, both having the same gender and age. SP training was conducted in three steps. In the first step, they practiced introducing themselves based on their script and conducting a question-and-answer portion. In the second step, they practiced the scenario algorithm, including the questions they were required to ask to cope with unexpected situations and provide feedback from the researcher. In the third step, two nursing students who do not participate in the study involved the final practice.

The participants in the PRP group received the same lecture and nursing skills practice. The group consisted of eight teams. Each team had four students that were equally assigned to two groups (two students per group) and conducted a similar practice simulation through PRP. While one group took care of the patient, one student in the other group served as the patient, while the other student recorded the group’s performance. Afterward, the two groups exchanged their roles. The control group did not receive orientation and debriefing for simulations; instead, they used the traditional hands-on training method. Each session took 120 min to complete. The program was conducted at different times and places to avoid exposing the participants to the program beforehand; that is, for the PRP group, role plays were conducted in the laboratory practice room during the afternoons, and for the Simulation group, simulations were conducted in two simulation rooms during the morning.

### 2.5. Instruments

#### 2.5.1. Knowledge of Standard Precaution

Knowledge of standard precaution was measured using the standard precautions knowledge measurement [38], which was developed by Cho and Choi [39] and modified based on the revised standard precautions model [6]. This tool included a total of 20 questions that were scored as 1 (if correct) or 0 (if incorrect). Participants’ score ranged from 0–20, with higher scores indicating higher knowledge of standard precautions. At the time of tool development, its reliability was not presented; in the present study, its Cronbach’s α value was 0.93.

#### 2.5.2. Awareness of Standard Precaution

The Awareness of Standard Precaution questionnaire, which was translated by Jeong [40] and developed by Hong et al. [41] based on the 2007 revised standard precautions model [6], was used to measure awareness of standard precautions. In our study, we included eight dimensions with a total of 36 items, after excluding two of the ten areas of the tool and their related questions: patient placement and infection control practices for special lumbar puncture procedure, which included ten questions for hand hygiene, three questions for respiratory etiquette, nine questions for personal protective equipment, two questions for patient care equipment, two questions for care of the environment, two questions for linen, five questions for safe injection practices, and three questions for worker safety. Each question was rated using a 5-point Likert scale ranging from 1 (“*not important”*) to 5 (“*very important*”), with a higher score indicating higher awareness. At the time of development, the tool reliability (Cronbach’s α) value was 0.95 [41], while it was 0.95 in this study.

#### 2.5.3. Infection-Related Anxiety

The measurement of infection-related anxiety felt during clinical practice was composed of items corresponding to the scenario based on the 2007 revised standard precaution [6]. The tool consisted of five questions in total, including anxiety related to infection due to blood exposure, body fluid exposure, patient-contact with those infected with respiratory diseases, injection puncture, and damaged skin contact according to the path of infection. The questions were rated using the Numeric Rating Scale, with the highest score of 10 showing “very extreme anxiety.” The higher the score, higher the anxiety. The Cronbach’s α value in this study was 0.97.

#### 2.5.4. Performance with Infection Control

Based on the scenario used in our study, we measured performance on infection control using a tool developed by the researcher, which is based on the 2007 revised standard precaution [6], Korea Centers for Disease Control and Prevention’s standard guidelines for preventing healthcare-related infections [15], and guidelines for tuberculosis treatment [42]. Among the eight dimensions of standard precaution, the dimensions related to hand hygiene, PPE, respiratory etiquette, safe injection practices, and worker safety were included. We excluded the dimensions on treatment, items, and linen, since nursing college students find them difficult to perform in clinical practice. The developed measurement was validated by two professors of fundamental nursing, two nursing faculties with experience in developing simulation scenarios, one clinical nurse with more than 10 years of clinical experience, and one medical doctor of infectious medicine (Content Validity Index: CVI). More than 80% of the items were finally selected. The measurement consisted of 15 questions: three on hand hygiene, six on PPE, two on safe injections, two on worker safety, and two on respiratory etiquettes; the scores ranged from 0 to 30, with higher scores indicating better ability to perform infection control. Two intensive care unit nurses who are unaware of the group assignment evaluated students’ performance by watching the video recording. The intra-class correlation coefficient was 0.96.

### 2.6. Data Collection

For the preliminary survey, information regarding the general characteristics, knowledge of standard precautions, awareness of standard precautions, and participants; infection-related anxiety were collected before the education method was implemented using a self-report questionnaire. For the post-doc survey, information regarding knowledge and awareness of standard precautions and data on infection-related anxiety were collected right after the education methods were conducted. Two intensive care unit nurses with more than three years of clinical experience evaluated the infection control performance using a video that was recorded on the day of the implementation of the education methods.

### 2.7. Data Analysis

The collected data were analyzed using the SPSS 23.0 statistical program (IBM Corp., Armonk, NY, USA). First, we compared the participants’ general characteristics between the simulation with SP group and PRP group using the chi-square test and Fisher’s exact test. Second, we used the Kolmogorov-Smirnov normality test for the main four variables. We used nonparametric tests to analyze three variables; knowledge, awareness of standard precaution, and infection-related anxiety that did not support normality. Third, we examined the three variables’ homogeneity between the two groups using the Mann-Whitney U test. Fourth, the groups were analyzed using the Mann-Whitney U test, and pre-post differences were tested using the Wilcoxon signed ranks test. Also, the difference in the infection control performance between the two groups was analyzed using a *t*-test. Regression analysis was not applicable since the variables did not satisfy the condition of normality.

### 2.8. Ethical Consideration

This study was conducted after receiving approval (IRB No: 1041078-201802-HR-037-01C) from C University. The subjects were informed of the study’s purpose, method, and confidentiality, including the nature of their voluntary participation and autonomy to withdraw from the study. All participants provided their written informed consent for the study.

## 3. Results

### 3.1. General Characteristics of Participants of the Two Groups

A total of 62 nursing students participated and were divided into two groups―29 in the experimental group and 33 in the control group. The participants were homogeneous in age, gender, average grade in the previous semester, simulation experience and the number of experiences, and educational experience for standard precautions (Table 2).

The average age was 21.41 (*SD* = 1.05) and 21.7 (*SD* = 1.45) years in the simulation using SP group and the PRP group, respectively. Regarding gender, most participants were female―24 in the simulation using SP group (82.8%) and 26 in the PRP group (78.8%). Regarding average grade last semester, 16 (55.2%) and 20 (60.6%) participants in the simulation using SP group and the PRP group, respectively, reported having a grade of 3.5 or higher. Regarding simulation experience, 23 (79.3%) and 20 (60.6%) participants have experienced simulation in the simulation using SP group and the PRP group, respectively. Regarding educational experience of standard precautions, 22 (75.9%) and 24 (72.7%) participants showed knowledge of standard precautions in the simulation using SP group and the PRP group, respectively. As for the educational method used for standard precautions, 7 (31.8%) and 13 (54.2%) participants reported using lecture only, 14 (63.6%) and 10 (41.7%) reported using lecture and practice classes, and 1 for both (4.5% and 4.2%) the simulation using SP group and PRP group, respectively, reported using simulation.

### 3.2. Homogeneity of the Variables between Two Groups

As a result of analyzing the prior homogeneity of the dependent variables between the simulation using SP group and the PRP group, we found no significant difference in knowledge of standard precautions (*z* = −0.1.194, *p* = 0.232), awareness of standard precautions (*z* = −1.425, *p* = 0.154), and infection-related anxiety (*z* = −0.304, *p* = 0.761) (Table 3).

### 3.3. Effects of Two Education Method between Two Groups

#### 3.3.1. Knowledge of Standard Precautions

The difference in knowledge of standard precautions score was 0.83 ± 1.34 and 0.61 ± 1.22 in the simulation using SP group and PRP group, respectively, with the simulation using SP group showing a greater degree of increase in knowledge. However, we found no statistically significant difference (*z* = −0.839, *p* = 0.401; see Table 4) between the two groups. Meanwhile, as a result of comparing the pre–post scores of each group, we found that their scores statistically increased from 17.79 ± 1.70 (pretest) to 18.62 ± 1.08 (posttest) in the simulation using SP group (*z* = −2.885, *p* = 0.004), and from 17.52 ± 1.20 (pretest) to 18.12 ± 0.99 (posttest) in the PRP group (*z* = −2.546, *p* = 0.011).

#### 3.3.2. Awareness of Standard Precautions

The difference in awareness of standard precautions score was 0.17 ± 0.40 and 0.29 ± 0.48 in the simulation using SP group and PRP group, respectively, with the PRP group showing a greater increase in degree of awareness. However, we found no statistically significant difference between the two groups (*z* = −0.1.304, *p* = 0.192; see Table 4). Meanwhile, as a result of comparing the pre–post scores of each group, we found that their scores statistically increased from 4.67 ± 0.30 (pretest) to 4.90 ± 0.19 (posttest) in the simulation using SP group (*z* = −4.178, *p* < 0.001), and from 4.57 ± 0.31 (pretest) to 4.87 ± 0.23 (posttest) in the PRP group (*z* = −4.628, *p* < 0.001).

#### 3.3.3. Infection-Related Anxiety

The difference in infection-related anxiety score was 3.00 ± 16.44 and −3.00 ± 15.11 in the simulation using SP group and PRP group, respectively, with no statistically significant difference between them (*z* = −1.468, *p* = 0.142; see Table 2). As a result of comparing the pre–post scores, we found that the scores of the PRP group decreased from 29.91 ± 9.83 (pretest) to 26.91 ± 11.83 (posttest) (z = −1.646, *p =* 0.100), while the scores of the simulation using SP group increased from 29.17 ± 9.95 (pretest) to 32.17 ± 11.19 (posttest) (*z* = −0.547, *p* = 0.605).

#### 3.3.4. Infection Control Performance

Regarding infection control performance, the simulation using SP group showed significantly higher scores (20.50 ± 3.03) than the PRP group (14.81 ± 3.04) (*z* = −7.354, *p* < 0.001). We found a statistically significant difference between the two in the subdomains of hand hygiene (*z* = −2.453, *p* = 0.017), PPE (*z* = −3.614, *p* = 0.001), respiratory etiquette (*z* = −5.032, *p* < 0.001), and worker safety (*z* = −4.005, *p* < 0.001) (Table 5).

## 4. Discussion

This study identified the effects of standard precautions infection education between two educational methods: simulation using SP and usual training using PRP. The education contents were organized to promote compliance with standard precautions, such as taking vital signs, blood sugar tests, subcutaneous injections, and wearing PPE, while carrying out nursing care. Taking vital signs is the most frequently used skill that nursing students must perform during clinical practice [43], while needlestick and sharps injuries from blood sugar tests and subcutaneous injections are the most common safety accidents among nursing college students [27,44]. It has been reported that injection-related accidents occur due to insufficient knowledge regarding blood-mediated infections and non-compliance with worker safety guidelines in standard precautions [27]. Meanwhile, previous studies have shown that there is low awareness and performance in wearing protective equipment in relation to compliance with standard precautions [18,23,45]; thus, it is considered as a skill that needs more practice opportunities. The nursing performances included in this program are the main nursing techniques of the core nursing skills [46] required for nursing students to improve their competencies upon graduating. Thus, improving these might lead to better competency in integrating knowledge and skills about infection control.

Based on our results, both education methods increased the knowledge of standard precautions significantly, with no significant difference between them. However, the participants in this study scored lower than those in previous studies involving nursing students in their third and fourth years regarding knowledge about standard precautions [23,41]. and higher than a study involving nursing students from all levels in Jordan, Taiwan, and Hong Kong regarding infection-related education [47,48,49]. The different scores in knowledge may be due to the level of participants in their undergraduate programs as well as the different nursing curriculum in other countries. The third year level students in this study were included at the beginning of the semester and have no experience of clinical practice. Hence, insufficient knowledge regarding clinical experience about standard precautions on infection control might influence our result. However, it is significant that the two education methods were useful in promoting knowledge. The simulation education with and without SP has reported greater improvements in knowledge compared to the usual PRP or lecture education [50,51]. Regarding PRP, our findings are similar to the results of previous studies showing that the method of peer-assisted learning is useful for improving the knowledge about standard precautions [52].

Furthermore, we believe that using the same lecture and scenario as in this study might lead to similar knowledge improvement when used in other settings. Indeed, a study [53] using the similar methods as in our study showed similar results. Moreover, in using lectures in practical education, the PRP method can be useful to acquire knowledge of standard precautions [31].

Awareness of standard precautions was improved in both simulation and PRP education. Prior studies on simulation-based infection control education reported that awareness was significantly increased during pre- and post-participation in the simulation [54]. In addition, a previous study reported that individuals in simulation-based infection control education improve more significantly in terms of awareness of standard precautions than those in conventional lecture-based training [55]. Meanwhile, the PRP method provides an opportunity to experience the roles of both care provider and patient [31]. The student is assimilated to the patient’s emotions while performing the patient’s role, leading to a more active engagement in practice [31,56]. Understanding the patient’s point of view regarding infection control would increase the awareness of standard precautions. Furthermore, using critical thinking and recognition as evaluators for peer efforts in solving problems together might increase awareness of standard precautions on infection control.

According to previous studies, awareness of standard precautions improves performance [18,57]. Considering the cost of simulation education [33,58], PRP methods can be a good alternative strategy for schools lacking financial support or a place for simulations [31]. Moreover, the safety environment influences the awareness and performance of standard precautions [18]. Thus, it is necessary to create a safe simulation environment that ensures patient safety and provides a safe place for learners that allow for friendly relationships with peers. Due to the lack of literature directly comparing the two education methods, further studies are needed to confirm our results on effective teaching methods for standard precautions on infection control.

In our study, participants’ anxiety level in the simulation using SP increased more than that of those in the PRP group. As reported in the previous studies [59,60], students might feel more nervous and uncomfortable in communicating with SP as well as during face-to-face interventions compared to peer practice. On the other hand, a systematic review study [61] reported that simulation practice decreased students’ anxiety before caring for a real patient. Simulation practice may increase the students’ anxiety during simulation but reduce it after simulation and make them feel lower than PRP practice before caring for a real patient. Interestingly, in previous studies involving hospital residents [31], the participants felt awkward about their patient role due to their previous relationship with their co-workers; moreover, their co-workers reacted more tolerantly when using medical terminology. In addition, the feeling of the actual situation was somewhat less in peer practice [31]. Therefore, to provide more realism and reduce anxiety when communicating with SP, the opportunities for simulation using SPs should be increased, and sufficient debriefing time between colleagues after simulation to share the feelings of anxiety and tension should be provided [62]. Future studies of interventions that help student better manage anxiety during simulation are needed.

The ability to perform infection control was higher among those in the group using SP. A study on safety skills reported that physiotherapy students using SP have higher confidence and preparedness regarding clinical placement in a workshop compared to those using PRP [63]. However, direct comparison is difficult due to the lack of prior studies comparing these two methods of infection control. Nevertheless, simulation programs on healthcare-related infection control education for nurses and nursing students have been reported to further improve participants’ performance than other educational methods, such as problem-based learning or lecture-centered education [33,55,64,65,66,67]. Simulation is an active learning method that involves students’ engagement; hence, it can be better and learning can be more lasting. This study shows that simulation-based education is more effective in cultivating clinical performance because it goes through the process of integrating knowledge while checking patient problems by simulating real-life situations and performing interventions directly. It can add additional evidence to the literature providing that simulation is an effective learning method. Moreover, debriefing during simulation is very useful in improving nursing knowledge and skills [62], and debriefing after PRP education will help improve performance.

This study has several limitations. First, since this study only extracted convenience samples from one university on a first-come, first-served basis, careful attention should be paid to the results’ generalization. Therefore, it is recommended that further studies that are conducted for verifying the effectiveness of infection control education programs consider involving a larger sample and adopting a randomized sampling. Second, this study compared the effects of two educational methods using only one scenario. To grasp the effect of practical training on infection control, further research that considers various scenarios and diverse learning methods is needed. Third, this study did not tackle the issue on cost-effectiveness related to education methods. Thus, further studies relating to cost-effectiveness will further determine the feasibility of these various educational methods.

Nevertheless, despite its limitations, it is worth noting that this is the first study that identified the effects of simulation using SP and PRP education, focusing on the effects of infection control, which is closely related to the safety of patients and students. Our findings can be used to develop various education programs that can be applied to practical training for knowledge and performance of infection control.

## 5. Conclusions

This study identified awareness of the standard precautions regarding infection control, infection-related anxiety, and performance in simulation using SP and PRP education. Although the two practical training methods differed in degree, nursing students’ knowledge and awareness of standard precautions increased in both training groups. The simulation using SP, however, was found to be more effective in improving nursing students’ infection control performance; therefore, it is recommended that simulation-based education be included in curricula focused on performance. Based on these results, it is suggested that nursing educators select the appropriate education method according to their practice environment and educational purposes, and develop more complicated scenarios for senior-level students. Further, the findings from the study can be used to develop education programs and integrated curricula to help graduate nurses prepare for professional practice and enhance their adherence to standard precautions in health care settings. Ultimately, future nurses who have been well-trained in infection control are expected to reduce infection rates and promote patient safety.

## Figures and Tables

**Table 1:** Education program design.

Simulation Using SP * Group		PRP * Group	
Contents	Time	Contents	Time
Orientation Lecture - Standard precautions definition - Guideline for standard precautions - Transmission-based precautions	50 min	Orientation Lecture - Standard precautions definition - Guideline for standard precautions - Transmission-based precautions	50 min
Skill Practice - Hand hygiene - PPE (N95 mask, gloves) * - Subcutaneous injection	30 min	Skill Practice - Hand hygiene - PPE (N95 mask, gloves) - Subcutaneous injection	30 min
Simulation Orientation	20 min	PRP Running - Each team consists of two groups (two students per group) - A group participates in the PRP - The other group serves as a patient and a recorder - Take turns participating in the PRP	40 min
Simulation Running - Each team consists of two groups (two students per group) - A group participates in the simulation - The other group observes simulations of the group. - Take turns participating in the simulation	40 min		
Debriefing	40 min		
Total time	180 min	Total time	120 min

* SP: standardized patient, PPE: personal protective equipment, PRP: peer role-play.

**Table 2:** General Characteristics of Participants of the two groups.

Characteristics		Simulation Using SP * (*n* = 29)	PRP * (*n* = 33)	χ2/*t*	*p*
		*M ± SD* or *n*(%)	*M ± SD* or *n*(%)		
Age		21.41 ± 1.05	21.70 ± 1.45	−0.642*	0.521
Gender	Female	24(82.8)	26(78.8)	0.156*	0.693
	Male	5(17.2)	7(21.2)		
Average Grade Point	<3.5	13(44.8)	13(39.4)	0.187*	0.665
	≥3.5	16(55.2)	20(60.6)		
Simulation experiences	Yes	23(79.3)	20(60.6)	2.541*	0.111
	No	6(20.7)	13(39.4)		
Number of simulation experience	One	2(8.7)	4(20.0)	1.326 ^a^	0.479
	Two	4(17.4)	4(20.0)		
	Three	17(73.9)	12(60.0)		
Standard precaution education experiences	Yes	22(75.9)	24(72.7)	0.079*	0.778
	No	7(24.1)	9(27.3)		
Educational experience of standard precautions	Lecture	7(31.8)	13(54.2)	2.384 ^a^	0.251
	Lecture with Practice	14(63.6)	10(41.7)		
	Simulation	1(4.5)	1(4.2)		

* SP: standardized patient, PRP: peer role-play, ^a^: Fisher’s exact test.

**Table 3:** Homogeneity of the variables between two groups.

Variables (No of Score)	Simulation Using SP * (*n* = 29)	PRP * (*n* = 33)	*z*	*p*
	*M ± SD*	*M ± SD*		
Knowledge of Standard Precaution (20)	17.79 *±* 1.70	17.52 *±* 1.20	−1.194	0.232
Awareness of Standard Precaution (5)	4.67 *±* 0.30	4.57*±* 0.31	−1.425	0.154
Infection-related Anxiety (50)	29.17 *±* 9.95	29.91*±* 9.83	−0.304	0.761

* SP: standardized patient, PRP: peer role-play.

**Table 4:** Difference of variables between two groups.

Variables	Simulation Using SP * (*n* = 29)			PRP * (*n* = 33)			*z*	*p*
	Pre	Post	Pre–Post	Pre	Post	Pre–Post		
	*M ± SD*	*M ± SD*	*M ± SD*	*M ± SD*	*M ± SD*	*M ± SD*		
Knowledge of standard precautions	17.79 ± 1.70	18.62 ± 1.08	0.83 ± 1.34	17.52 ± 1.20	18.12 ± 0.99	0.61 ± 1.22	−0.839	0.401
Awareness of standard precautions	4.67 ± 0.30	4.90 ± 0.19	0.17 ± 0.40	4.57 ± 0.31	4.87 ± 0.23	0.29 ± 0.48	−1.304	0.192
Infection-related Anxiety	29.17 ± 9.95	32.17 ± 11.19	3.00 ± 16.44	29.91 ± 9.83	26.91 ± 11.83	−3.00 ± 15.11	−1.468	0.142

* SP: standardized patient, PRP: peer role-play.

**Table 5:** Comparison of Infection Control Performance after Education (*N* = 62).

Variables	Simulation Using SP * (*n* = 29)	PRP * (*n* = 33)	*t*	*p*
	*M ± SD*	*M ± SD*		
Infection Control Performance	20.50 ± 3.03	14.81 ± 3.04	−7.354	<0.001
Hand hygiene	2.21 ± 1.77	1.21 ± 1.42	−2.453	0.017
PPE *	8.60 ± 1.89	6.48 ± 2.70	−3.614	0.001
Respiratory etiquette	2.76 ± 0.85	1.52 ± 1.06	−5.032	<0.001
Safe injection practices	3.79 ± 0.56	3.70 ± 0.68	−0.601	0.550
Worker safety	3.14 ± 1.05	1.91 ± 1.33	−4.005	<0.001

* SP: standardized patient, PRP: peer role-play, PPE: personal protective equipment.

## Data Availability

The data presented in this study are available on request from the corresponding author. The data are not publicly available due to restrictions eg privacy or ethical.

## References

[1] Allegranzi B., Nejad S.B., Combescure C., Graafmans W., Attar H., Donaldson L., Pittet D. (2011). Burden of endemic health-care-associated infection in developing countries: Systematic review and meta-analysis. Lancet.

[2] Marchetti A., Rossiter R. (2013). Economic burden of healthcare-associated infection in US acute care hospitals: Societal perspective. J. Med. Econ..

[3] World Health Organization (WHO) (2020). Director-General’s Remarks at the Media Briefing on 2019-nCoV. https://www.who.int/dg/speeches/detail/who-director-general-s-remarks-at-the-media-briefing-on-2019-ncov-on-11-february-2020.

[4] World Health Organization (2004). Practical Guidelines for Infection Control in Health Care Facilities. https://apps.who.int/iris/handle/10665/206946.

[5] Joukar F., Mansour-Ghanaei F., Soati F., Meskinkhoda P. (2012). Knowledge levels and attitudes of health care professionals toward patients with hepatitis C infection. World J. Gastroenterol..

[6] Siegel J.D., Rhinehart E., Jackson M., Chiarello L. (2007). 2007 Guideline for Isolation Precautions: Preventing Transmission of Infectious Agents in Health Care Settings. Am. J. Infect. Control..

[7] Centers for Disease Control and Prevention (2020). Interim Infection Prevention and Control Recommendations for Healthcare Personnel during the Coronavirus Disease 2019 (COVID-19) Pandemic. https://www.cdc.gov/coronavirus/2019-ncov/hcp/infection-control-recommendations.html.

[8] Asmr Y., Beza L., Engida H., Bekelcho T., Tsegaye N., Aschale Y. (2019). Assessment of Knowledge and Practices of Standard Precaution against Blood Borne Pathogens among Doctors and Nurses at Adult Emergency Room in Addis Ababa, Ethiopia. Emerg. Med. Int..

[9] Donati D., Biagioli V., Cianfrocca C., De Marinis M.G., Tartaglini D. (2019). Compliance with Standard Precautions among Clinical Nurses: Validity and Reliability of the Italian Version of the Compliance with Standard Precautions Scale (CSPS-It). Int. J. Environ. Res. Public Health.

[10] Gammon J., Morgan-Samuel H., Gould D. (2007). A review of the evidence for suboptimal compliance of healthcare practitioners to standard/universal infection control precautions. J. Clin. Nurs..

[11] Mohd-Nor N., Bit-Lian Y. (2019). Knowledge, Attitude and Practices of Standard Precaution among Nurses in Middle-East Hospital. SciMedicine J..

[12] Danzmann L., Gastmeier P., Schwab F., Vonberg R.-P. (2013). Health care workers causing large nosocomial outbreaks: A systematic review. BMC Infect. Dis..

[13] Hessels A.J., Larson E. (2016). Relationship between patient safety climate and standard precaution adherence: A systematic review of the literature. J. Hosp. Infect..

[14] World Health Organization (2020). Infection Prevention and Control during Health Care when Covid-19 is Suspected: Interim Guidance. https://www.who.int/publications/i/item/10665-331495.

[15] The Korea Centers for Disease Control and Prevention Standard Guidelines for Preventing Healthcare-Related Infections 2017. https://m.blog.naver.com/PostView.nhn?blogId=tinaarena&logNo=221144257694&proxyReferer=https:%2F%2Fwww.google.com%2F.

[16] Moralejo D., El Dib R., Prata R.A., Barretti P., Corrêa I. (2018). Improving adherence to Standard Precautions for the control of health care-associated infections. Cochrane Database Syst. Rev..

[17] Zhang Y., Sun Z., Latour J.M., Hu B., Qian J. (2020). Hospital response to the COVID-19 outbreak: The experience in Shanghai, China. J. Adv. Nurs..

[18] Cha J.-E., Cho J., Kim Y.-G., Nam G.-H., Lee S., Lee S.-Y., Lee A., Lee J., Chae S. (2017). Nursing student safety-climate, perception and performance of standard precautions for healthcare-associated infection control. J. Korean Acad. Ind. Coop. Soc..

[19] Park J.H., Kim H.S. (2012). The Effect of the Hand Hygiene Education Program on Hand Hygiene Knowledge, Hand Hygiene Perception, Nasal Staphylococcus aureus Colonization and Hand Hygiene Adherence in Nursing Students. J. Korean Biol. Nurs. Sci..

[20] Magill S.S., O’Leary E., Janelle S.J., Thompson D.L., Dumyati G., Nadle J., Wilson L.E., Kainer M.A., Lynfield R., Greissman S. (2018). Changes in Prevalence of Health Care–Associated Infections in U.S. Hospitals. New Engl. J. Med..

[21] Ward D.J. (2011). The role of education in the prevention and control of infection: A review of the literature. Nurse Educ. Today.

[22] Al-Hussami M., Darawad M. (2013). Compliance of nursing students with infection prevention precautions: Effectiveness of a teaching program. Am. J. Infect. Control..

[23] Jeong M.-H. (2015). Survey of Exposure to Blood and Body Fluids, Knowledge, Awareness and Performance on Standard Precautions of Infection Control in Nursing Students. J. Korea Contents Assoc..

[24] Kim J.-M., Choi Y.-S. (2015). Effect of practice education using the simulator, critical thinking, problem solving ability and nursing process confidence of nursing students. J. Digit. Converg..

[25] Weinberg E.R., Auerbach M.A., Shah N.B. (2009). The use of simulation for pediatric training and assessment. Curr. Opin. Pediatr..

[26] Kim M.Y., Kim M., Kim J., Maeng J., Park S., Son J. (2017). Perception and Inner Struggle Experienced by Nursing Students in Relation with Infection Management through Observation and Performance of Infection Control Activities. Perspect. Nurs. Sci..

[27] Seo J.H., Jung E.Y. (2017). Factors influencing nursing students’ performance on standard precautions of infection control. J. Korean Biol. Nurs. Sci..

[28] Cant R.P., Cooper S.J. (2010). Simulation-based learning in nurse education: Systematic review. J. Adv. Nurs..

[29] Cook D.A., Hatala R., Brydges R., Zendejas B., Szostek J.H., Wang A.T., Erwin P.J., Hamstra S.J. (2011). Technology-Enhanced Simulation for Health Professions Education: A Systematic Review and Meta-analysis. JAMA.

[30] Lewis K.L., Bohnert C.A., Gammon W.L., Hölzer H., Lyman L., Smith C., Thompson T.M., Wallace A., Gliva-McConvey G. (2017). The association of standardized patient educators (ASPE) standards of best practice (SOBP). Adv. Simul..

[31] Paramasivan A., Khoo D. (2020). Standardized Patients Versus Peer Role Play—Exploring the Experience, Efficacy, and Cost-Effectiveness in Residency Training Module for Breaking Bad News. J. Surg. Educ..

[32] Nestel D., Tabak D., Tierney T., Layat-Burn C., Robb A., Clark S., Morrison T., Jones N., Ellis R., Smith C. (2011). Key challenges in simulated patient programs: An international comparative case study. BMC Med. Educ..

[33] Luctkar-Flude M., Baker C., Hopkins-Rosseel D., Pulling C., McGraw R., Medves J., Krause A., Brown C.A. (2014). Development and Evaluation of an Interprofessional Simulation-Based Learning Module on Infection Control Skills for Prelicensure Health Professional Students. Clin. Simul. Nurs..

[34] Faul F., Erdfelder E., Buchner A., Lang A.-G. (2009). Statistical power analyses using G* Power 3.1: Tests for correlation and regression analyses. Behav. Res. Methods.

[35] Kim H.-S. (2009). Effect of infection control education on knowledge, attitude and self-confidence of student nurses about nosocomial infection control. J. Korean Soc. Health Educ Promot..

[36] Kim Y., Kim M.Y., Seo Y.H. (2016). The Effects of an Intensive Education Program on Hospital Infection Control on Nursing Students’ Knowledge, Attitude, and Confidence in Infection Control. J. Korean Biol. Nurs. Sci..

[37] Fanning R.M., Gaba D.M. (2007). The Role of Debriefing in Simulation-Based Learning. Simul. Health J. Soc. Simul. Healthc..

[38] Oh J.-Y., Mun J.-Y., Oh H.-K. (2016). Affecting Factors on Performance of Nursing Students regarding Standard Precautions for Healthcare associated Infection Control and Prevention. J. Health Inform. Stat..

[39] Cho G.L., Choi J.S. (2010). Knowledge of and compliance with standard precautions by nurses in intensive care unit. J. Korean Acad. Fundam. Nurs..

[40] Jeong S. Standard precaution. Proceedings of the Korean Society for Nosocomial Infection Control 13th Conference.

[41] Hong S.-Y., Kwon Y.-S., Park H.-O. (2012). Nursing Students’ Awareness and Performance on Standard Precautions of Infection Control in the Hospital. J. Korean Acad. Soc. Nurs. Educ..

[42] Korea Centers for Disease Control and Prevention (2017). Korean Guidelines for Tuberculosis.

[43] Han H.-H. (2016). A Study of Convergence on Frequency of Performance, Self-Confidence, Performance Assessment Scores of Core Nursing Skills of Nursing Students. J. Digit. Converg..

[44] Moon M.-Y. (2018). A study on knowledge, attitude and confidence in performance of patient safety with experience of safety incidents during clinical practicum in nursing students. AJMAHS.

[45] Lee S.J., Park J.-Y., Jo N. (2017). Influence of Knowledge and Awareness on Nursing Students’ Performance of Standard Infection Control Guidelines. J. Korean Acad. Nurs. Adm..

[46] Korean Accreditation Board of Nursing Education (2015). Certification Criteria and Handbook. http://www.kabone.or.kr/kabon02/index04.php.

[47] Darawad M.W., Al-Hussami M. (2013). Jordanian nursing students’ knowledge of, attitudes towards, and compliance with infection control precautions. Nurse Educ. Today.

[48] Tafazoli M., Javidi-Sarafan M., Khadivzadeh T., Mazloum S.R. (2020). Comparing the effect of standardized patient-based education and feedback lecture on midwives’ knowledge and practice in counseling screening for fetal malformations. J. Educ. Health Promot..

[49] Wu C.-J., Gardner G., Chang A.M. (2009). Nursing students’ knowledge and practice of infection control precautions: An educational intervention. J. Adv. Nurs..

[50] Byun H.-S., Kwon K.-H., Suh B.-D. (2014). Effect of a Simulation-based Education for Advanced Cardiovascular Life Support on Knowledge, Self-efficacy, Clinical Performance Ability and Problem Solving Process in Nursing Students. J. Korea Entertain. Ind. Assoc..

[51] Kim H.-Y., Kim H.-R. (2015). Effects of a Colonoscopy based Simulation Education Program on Knowledge and Clinical Performance in Nursing Students. Korean J. Adult Nurs..

[52] Desnita R., Surya D.O. (2020). Effectiveness of Peer-Assisted Learning in Nursing Student Knowledge and Compliance in The Application of Standard Precautions. J. Keperawatan Indones..

[53] Jang K.-I. (2013). Development and Effects of an Oncology Nursing Simulation Program for Nursing Students. Ph.D. Thesis.

[54] Cho H.-Y. (2015). Effect of Cooperative Learning Applying Jigsaw Model in Simulation-Based Infection Control Education on Perception of Infection Control, Intrinsic Motive and Learning Satisfaction. J. Korea Acad. Cooperat. Soc..

[55] Cho S.S., Kim K.M., Lee B.Y., Park S.A. (2012). The effects of simulation-based infection control training on the intensive care unit nurses’ perception, clinical performance, and self-efficacy of infection control. J. Korean Clin. Nur. Res..

[56] Jackson V.A., Back A.L. (2011). Teaching Communication Skills Using Role-Play: An Experience-Based Guide for Educators. J. Palliat. Med..

[57] Park S.H., Byun E.K. (2020). Effects of self-efficacy, standard precaution knowledge, awareness on performance of nursing students. J. Korea Acad. Ind. Coop. Soc..

[58] Patrício M.F., Julião M., Fareleira F., Carneiro A.V. (2013). Is the OSCE a feasible tool to assess competencies in undergraduate medical education?. Med. Teach..

[59] Slater L.Z., Bryant K.D., Ng V. (2016). Nursing Student Perceptions of Standardized Patient Use in Health Assessment. Clin. Simul. Nurs..

[60] Lee S.J., Kim S.S., Park Y.-M. (2015). First experiences of high-fidelity simulation training in junior nursing students in Korea. Jpn. J. Nurs. Sci..

[61] Labrague L.J., McEnroe-Petitte D.M., Bowling A.M., Nwafor C.E., Tsaras K. (2019). High-fidelity simulation and nursing students’ anxiety and self-confidence: A systematic review. Nurs. Forum.

[62] Kim S.S., De Gagne J.C. (2018). Instructor-led vs. peer-led debriefing in preoperative care simulation using standardized patients. Nurse Educ. Today.

[63] Phillips A.C., Mackintosh S., Bell A., Johnston K. (2017). Developing physiotherapy student safety skills in readiness for clinical placement using standardised patients compared with peer-role play: A pilot non-randomised controlled trial. BMC Med. Educ..

[64] Kim S. (2016). Development and Effects of a Simulation-Based Education Program for Healthcare-Associated Infection Control for Nursing Students. Ph.D. Thesis.

[65] Kim Y.-E., Kang H.-Y. (2013). Development and Application of Simulation Learning Scenario using Standardized Patients: Caring for Neurological Patients in Particular. J. Korea Contents Assoc..

[66] Kim Y.A., Yoon S. (2018). The effects of nursing practical education using standardized patients in Korea: A systematic review and meta-analysis. J. Korean Data Inf. Sci. Soc..

[67] Nakamura I., Fujita H., Tsukimori A., Kobayashi T., Sato A., Fukushima S., Amano K., Abe Y. (2019). Scenario-based simulation health care education for performance of hand hygiene. Am. J. Infect. Control..

